# *Cannabis sativa*-based oils against aluminum-induced neurotoxicity

**DOI:** 10.1038/s41598-023-36966-9

**Published:** 2023-06-17

**Authors:** Carla Alves, Wagner Antonio Tamagno, Ana Paula Vanin, Aline Pompermaier, Leonardo José Gil Barcellos

**Affiliations:** 1 Biochemistry and Molecular Biology Laboratory Rosilene Rodrigues Kaizer, Instituto Federal de Educação, Ciência e Tecnologia do Rio Grande do Sul, Campus Sertão, Sertão, RS Brazil; 2 Bioexperimentation Graduate Program, Universidade de Passo Fundo, Passo Fundo, RS Brazil; 3 Pharmacology Graduate Program, Universidade Federal de Santa Maria, Santa Maria, RS Brazil; 4 Civil and Environmental Engineering Graduate Program, Universidade de Passo Fundo, Passo Fundo, RS Brazil

**Keywords:** Enzyme mechanisms, Enzymes, Lipids, Metals, Neurochemistry

## Abstract

The use of terpenoid compounds in different neural-related conditions is becoming useful for several illnesses. Another possible activity of these compounds is the reduction of nervous impairment. *Cannabis sativa* plants are known for their concentration of two important terpenoids, the delta-9-tetrahydrocannabinol (THC) and cannabidiol (CBD). CBD and THC have central peripheral activities already described and their usage in different brain diseases, such as Alzheimer's and multiple sclerosis. Aluminum (Al) is known as an important neurotoxic compound, the physiological action of Al is not known already, and in high concentrations can lead to intoxication and cause neurotoxicity. Here we evaluated the potential effect of two different doses of CBD- and THC-rich based oils against Al-induced toxicity, in the zebrafish model. We evaluated behavioral biomarkers of the novel tank test (NTT) and social preference test (SPT), and biochemical markers: the activity of the enzyme acetylcholinesterase (AChE) and the antioxidant enzymes—catalase, superoxide dismutase, and glutathione-S-transferase. CBD- and THC-based oils were able to increase the AChE activity helping the cholinergic nervous system actuate against Al toxicity which was reflected by the behavioral biomarkers changes. We concluded that the oils have a protective effect and might be used with proposals for neurological and antioxidant impairment avoidance caused by Al intoxications.

## Introduction

*Cannabis sativa* has been used for a long time in the treatment of neurodegenerative diseases, anxiety-like conditions, autistic spectrum disorder, and others^1–3^. Among the hundreds of chemical compounds produced by the secondary metabolism of *C. sativa*, some of them are compounds with biological activity, such as terpenes and phenolic compounds^4^. Two important terpenoids produced by this plant and considered target compounds for medical utilization are the delta-9-tetrahydrocannabinol (THC) and cannabidiol (CBD)^5^.

THC and CBD stand out as the two main cannabinoids^6^. THC is nicely comprehended for its psychoactive effects, while CBD is a non-psychostimulant cannabinoid^7^. The mechanisms responsible for most CBD and THC effects are still not completely clear. But it is known that these compounds interact with different systems modulating them, like the cholinergic nervous system (CholNS)^8^, endocannabinoid system^9^, serotoninergic nervous system, and on neurotransmitter content^10^. These interactions affect the development, synaptic malleability, and reaction to endogenous and environmental injury, as a feature of considerable neurodegenerative disorders^11^. Via the interchange between cannabinoids and their receptors, an anti-inflammatory reaction is developed^12^, which is a crucial strategy for supporting physiological homeostasis and working on the central nervous system, including discomfort decrease^13^.

The CholNS is an important system due to its responsibility for the maintenance of cognition, memory, movement, and environment perception^14^**.** The neurotransmitter of the CholNS is acetylcholine (ACh). ACh is an excitatory neurotransmitter constructed from acetyl coenzyme-A and choline, which act at neuromuscular connections and regions of the brain linked to learning^15^. Thus, the CholNS changes are, hence, involved in neurodegenerative disorders such as Parkinson's and Alzheimer's diseases (AD)^16–18^. In cholinergic signaling, the enzyme acetylcholinesterase (AChE) ends the nerve stimulation via the hydrolysis of ACh into acetate and choline^19^. revealed that patients with AD were enhanced when they were feted with AChE inhibitors. Equal studies documented that acute CBD exposition induces substantial alterations in brain activity during resting states and performance of cognitive tasks in both healthy recruits and patients with psychiatric illnesses^20^. The security of CBD was also tried in *Caenorhabditis elegans* and no toxicity results were observed^21^.

CholNS is largely affected by different toxicants such as pesticides^22^, toxins^23^, and metals such as aluminum (Al)^15,24,25^. Al is the third most abundant metal (8%) present on the surface of the earth it is not known physiological function of this metal in mammalians^26^**.** Al is considered an oxidant and neurotoxic compound due to its ability to bioaccumulate in the body tissue and react with the cell compounds generating oxidative stress conditions^27^. Due to Al's abundance, it is largely used in the pharmaceutical, cosmetic, and metallurgical industries, as well as in the production of Al alloys that are intensely used in the making of domestic utilitarian objects^26^. This intense use increases the exposition of the Al and consequently its contamination.

Due to the ability of Al to be considered an oxidant compound, it is common the observation of a high generation of reactive oxygen species (ROS) in the cells. However, Al might interact with several antioxidant enzymes such as superoxide dismutase (SOD) and catalase (CTL) which are metalloenzymes that contain a metallic nucleus and is easily modulated by Al^28–30^. The antioxidant system is important in the re-establishment of oxidative homeostasis, on the contrary, may increase oxidative stress conditions and lead to cell death, senile aging, and disease decompensation.

Nowadays, some compounds are known as Al chelating such as deferoxamine (DFX)^30,31^. Because of that, in this study, we aimed to evaluate the neural protection of two different *C. sativa*-based oils named THC- and CBD-rich oils on zebrafish exposed to Al (5.5 mg/L). To assess this, we evaluated behavioral biomarkers of anxiety-like conditions as well as social behavior, two key behaviors on zebrafish neurotoxicity evaluation. In addition, due to Al's interaction with CholNS and the antioxidant system, we evaluated the effect of the oils on AChE activity as the marker of CholNS function and the antioxidant enzymes SOD, CTL, and glutathione S-transferase (GST). Based on the evaluation of these biomarkers we aimed to understand whether or not the THC- and/or CBD-based oils might have a neuroprotector effect against Al toxicity.

## Results

### Delta-9-tetrahydrocannabinol (THC) and cannabidiol (CBD) quantification in the exposition water

**Figure 1:**
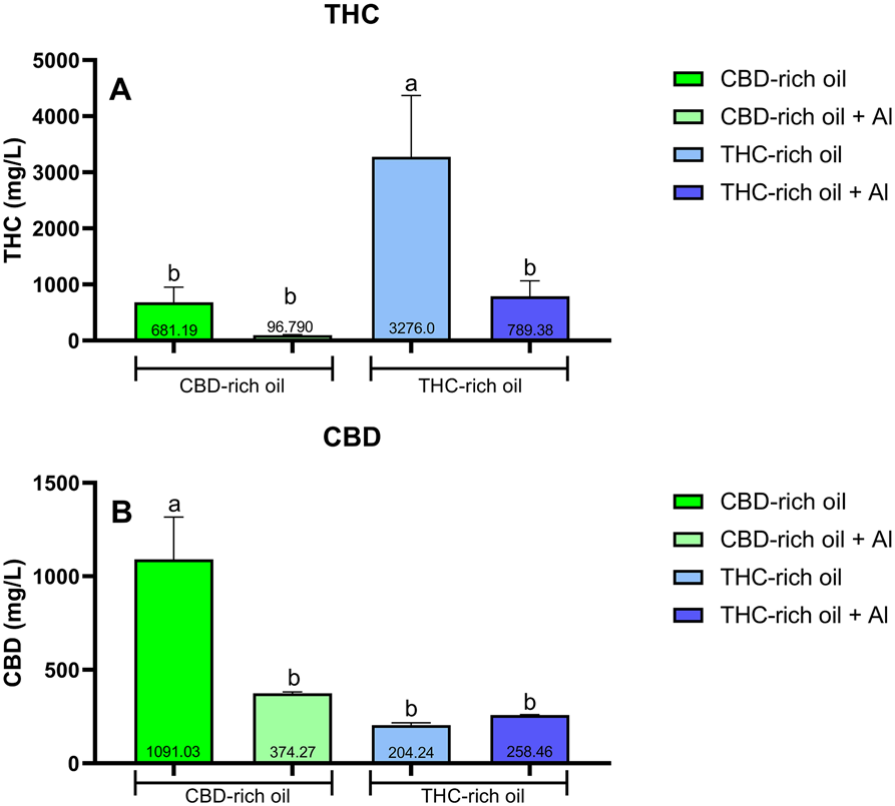
- HPLC quantification of THC (**A**) and CBD (**B**) on water samples destinated for fish exposure. Data are presented as mean ± SEM and analyzed by one-way ANOVA followed by Tukey’s multiple range test. The same lower-case letters represent no statistically significant differences among the groups.

The concentration of THC and CBD in the water of the tanks for the fish exposition was quantified even in the presence and absence of Al. THC (Fig. 1A) was present in both cannabis oils even though in a higher concentration in the THC-rich oil. The concentration of the major compound THC in both oils looks to be decreased when in the presence of Al. The quantification of the compound CBD (Fig. 1B) observed its presence in both cannabis oils however the presence of CBD was higher in CBD-rich oil without Al. Here once more the presence of Al decreased the concentration of CBD.

In the other exposition groups that are not presented in Fig. 1 (vehicle and vehicle + Al) the concentrations of CBD and THC were not detected.

### Behavioral analyzes

#### Novel Tank Test (NTT)

**Figure 2:**
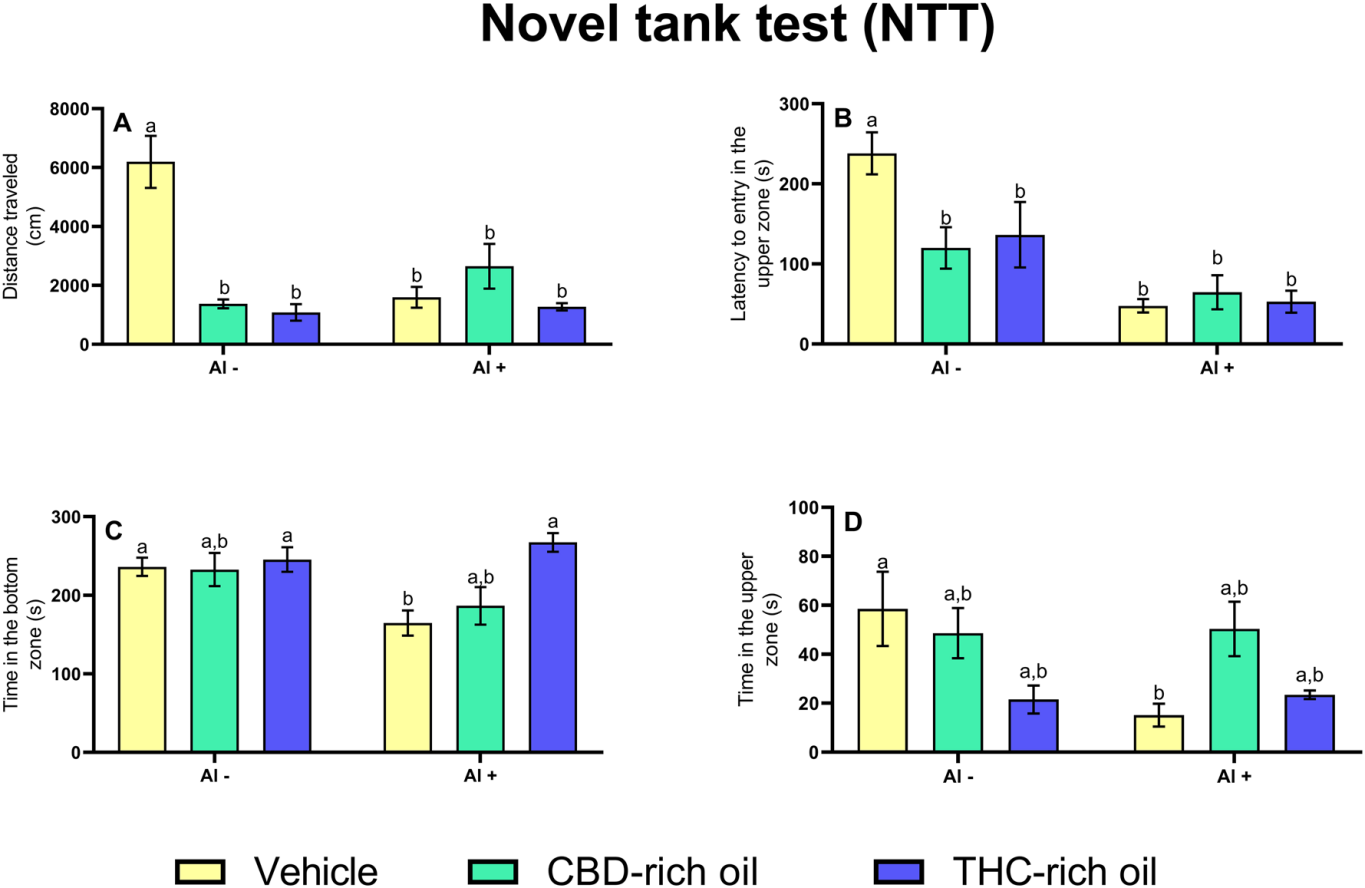
Novel Tank Test (NTT) of zebrafish exposed to different terpenoid-rich oils and Al. Evaluations of distance traveled (**A**), latency to entry in the upper zone (**B**), time in the bottom zone (**C**), and time in the upper zone (**D**). Data are presented as mean ± SEM analyzed by two-way ANOVA followed by Tukey’s multiple range test. The same lower-case letters represent no statistically significant differences among the groups.

The distance traveled (Fig. 2A) was increased in the vehicle-Al- in comparison to the CBD-rich oil-Al- (*p *< 0.0001) THC-rich oil-Al- (*p *< 0.0001), vehicle-Al + (*p *< 0.0001), CBD-rich oil-Al + (*p *< 0.001), and THC-rich oil-Al + (*p *< 0.0001). The latency to entry in the upper zone (Fig. 2B) was increased in the vehicle-Al- in comparison to the CBD-rich oil-Al- (*p *< 0.05), THC-rich oil-Al- (*p *< 0.05), vehicle-Al + (*p *< 0.0001), CBD-rich oil-Al + (*p *< 0.0001), and THC-rich oil-Al + (*p *< 0.0001). The time in the bottom zone (Fig. 2C) was decreased in the vehicle-Al + in comparison to vehicle-Al- (*p *< 0.05), TCH-rich oil-Al- (*p *< 0.05), and THC-rich oil-Al + (*p *< 0.001). Time in the upper zone (Fig. 2D) was decreased in the group treated with vehicle-Al + in comparison to vehicle-Al- (*p *< 0.05).

#### Social preference test (SPT)

**Figure 3:**
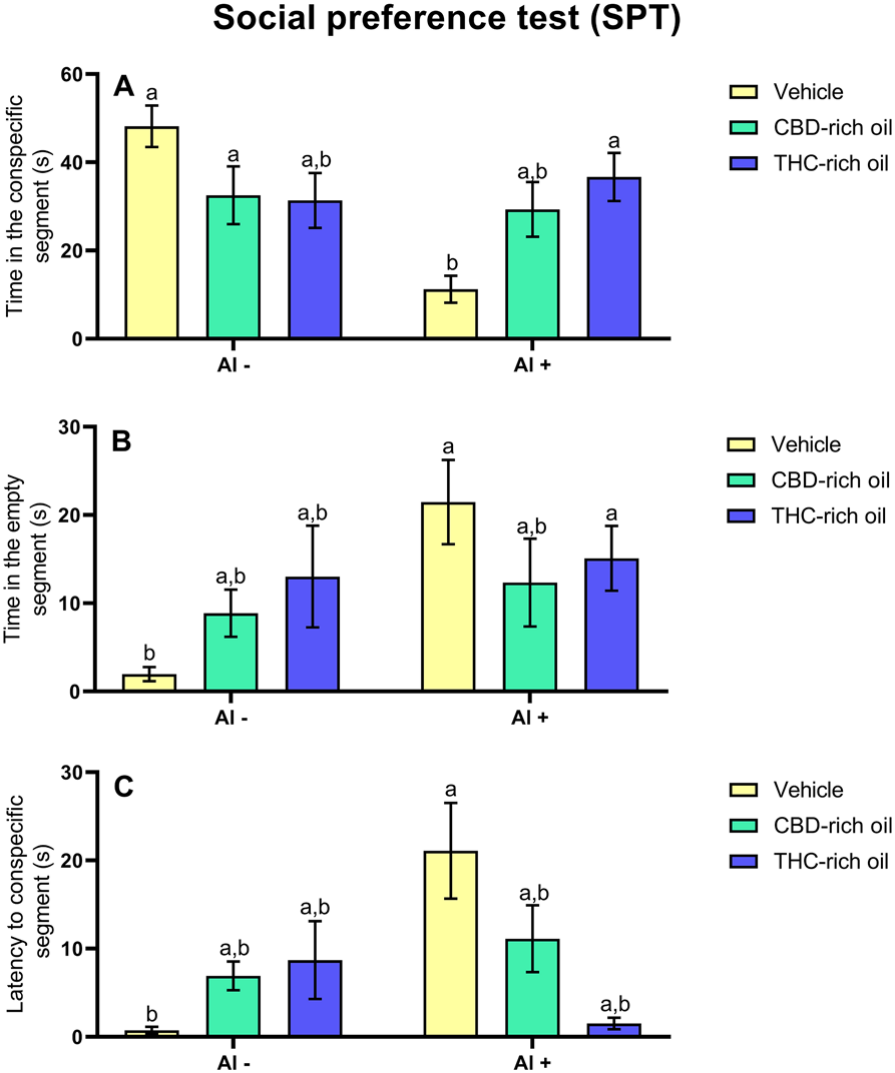
Social Preference Test (SPT) of zebrafish exposed to different terpenoid-rich oils and Al. Evaluations of time in the conspecific segment (**A**), time in the empty segment (**B**), and latency to the conspecific segment (**C**). Data are presented as mean ± SEM and analyzed by two-way ANOVA followed by Tukey’s multiple range test. The same lower-case letters represent no statistically significant differences among the groups.

The time in the conspecific segment (Fig. 3A) was decreased in the group treated with Vehicle-Al + in comparison to vehicle-Al- (*p *< 0.001), CBD-rich oil-Al- (*p *< 0.05), and THC-rich oil + Al + (*p *< 0.001). In the time in the empty segment (Fig. 3B), the group vehicle-Al- had a significant decrease in the time in comparison to vehicle-Al + (*p *< 0.001) and THC-rich oil-Al + (*p *< 0.05). In the latency to a conspecific segment (Fig. 3C), the group treated with vehicle-Al + had a significant increase in the latency time in comparison to vehicle-Al- (*p *< 0.05).

### Biochemical analyzes

#### Acetylcholinesterase activity

**Figure 4:**
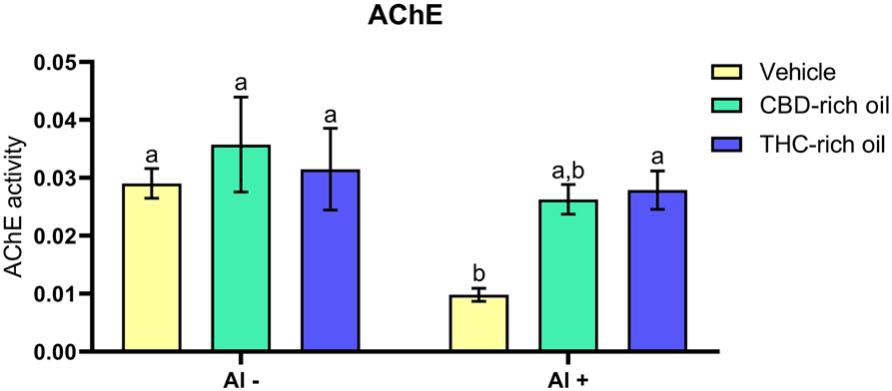
Acetylcholinesterase activity (AChE) of zebrafish exposed to different terpenoid-rich oils and Al. AChE activity was expressed as µmol of ACh/h/mg of protein. Data are presented as mean ± SEM and analyzed by two-way ANOVA followed by Tukey’s multiple range test. The same lower-case letters represent no statistically significant differences among the groups.

AChE activity (Fig. 4) was decreased in the group treated with Al (vehicle-Al +) in comparison to vehicle-Al- (*p *< 0.05), CBD-rich oil; Al- (*p *< 0.001), THC-rich oil: Al- (*p *< 0.05), and THC-rich oil-Al + (*p *< 0.05).

#### Superoxide dismutase activity

**Figure 5:**
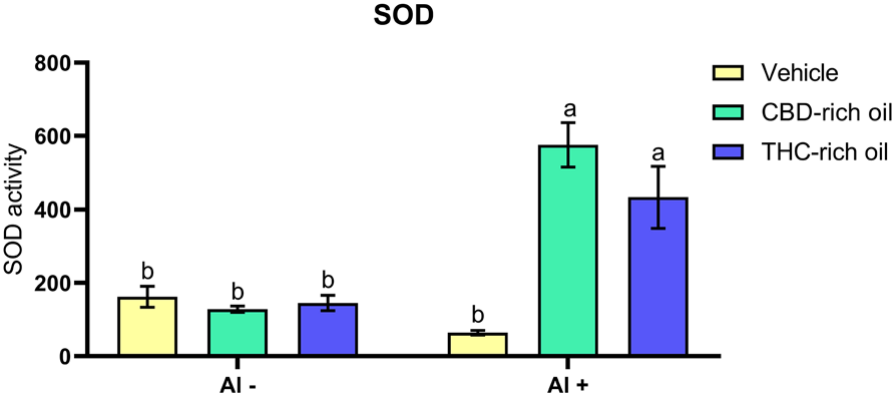
Superoxide dismutase activity (SOD) of zebrafish exposed to different terpenoid-rich oils and Al. SOD activity was expressed as U of SOD/mg of protein. Data are presented as mean ± SEM and analyzed by two-way ANOVA followed by Tukey’s multiple range test. The same lower-case letters represent no statistically significant differences among the groups.

SOD activity (Fig. 5) was largely increased in the groups treated with Al and CBD-rich oil/THC-rich oil in comparison to vehicle-Al- (*p *< 0.0001; *p *< 0.001), CBD-rich oil-Al- (*p *< 0.0001; *p *< 0.0001, respectively), THC-rich oil-Al- (*p *< 0.0001; *p *< 0.001, respectively) and vehicle-Al + (*p *< 0.0001; *p *< 0.0001, respectively).

#### Catalase activity

**Figure 6:**
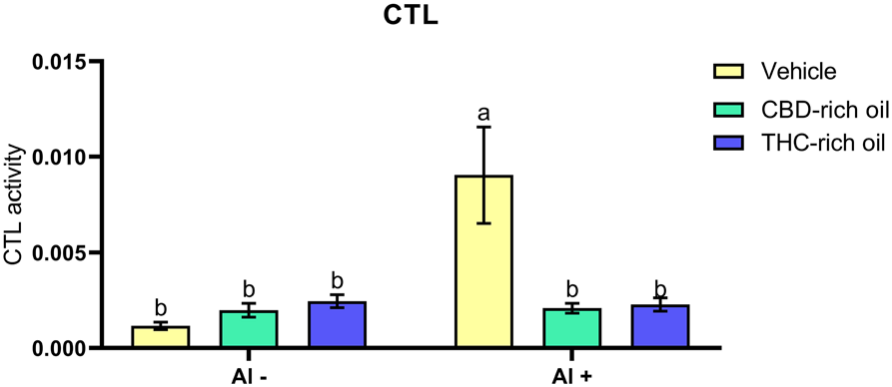
Catalase activity (CTL) of zebrafish exposed to different terpenoid-rich oils and Al. CTL activity was expressed as μmol H_2_O_2_/mg protein/min. Data are presented as mean ± SEM evaluated and analyzed by two-way ANOVA followed by Tukey’s multiple range test. The same lower-case letters represent no statistically significant differences among the groups.

The CTL activity (Fig. 6) was increased in the group treated with vehicle-Al + in comparison to vehicle-Al- (*p *< 0.0001), CHD-rich oil-Al- (*p *< 0.001), THC-rich oil-Al- (*p *< 0.001), CBD-rich oil-Al + (*p *< 0.001), and THC-rich oil-Al + (*p *< 0.001).

#### Glutathione-S-transferase activity

**Figure 7:**
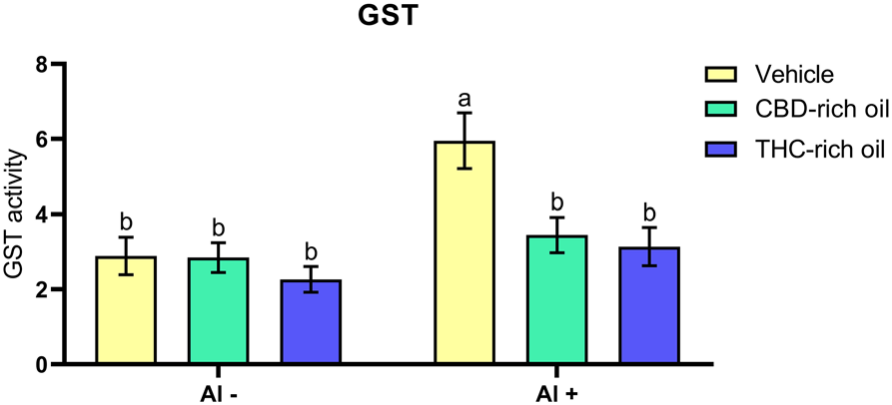
Glutathione-S-transferase activity (GST) of zebrafish exposed to different terpenoid-rich oils and Al. GST activity was expressed as μmol subtract acted/mg protein/min. Data are presented as mean ± SEM and analyzed by two-way ANOVA followed by Tukey’s multiple range test. The same lower-case letters represent no statistically significant differences among the groups.

The GST activity (Fig. 7) was increased in the group treated with vehicle-Al + in comparison to vehicle-Al- (*p *< 0.001), CHD-rich oil-Al- (*p *< 0.001), THC-rich oil-Al- (*p *< 0.0001), CBD-rich oil-Al + (*p *< 0.01), and THC-rich oil-Al + (*p *< 0.01).

All statistical details are depicted in Table 1.

**Table 1:** Statistics details of the testes.

Test	Parameter	Figure	Statistical value (DF)	*P*-value
NTT	Distance traveled	2A	F _2, 93_ = 16.14	< 0.0001
	Latency to the upper one	2B	F _2, 60_ = 5.006	< 0.01
	Time in the bottom zone	2C	F _2, 78_ = 4.102	< 0.05
	Time in the upper zone	2D	F _2, 90_ = 3.847	< 0.05
SPT	Time in the conspecific segment	3A	F _2, 72_ = 8.255	< 0.001
	Time in the empty segment	3B	F _2, 72_ = 2.758	> 0.05
	Latency to conspecific segment	3C	F _2, 72_ = 8.632	< 0.005
Biochemical biomarkers	AChE	4	F _2, 159_ = 1.444	> 0.05
	SOD	5	F _2, 98_ = 19.59	< 0.0001
	CTL	6	F _2, 204_ = 8.293	< 0.001
	GST	7	F _2, 154_ = 3.364	< 0.05

## Discussion

Here we show that fish exposed to Al presented an anxiety-like behavioral phenotype with reduced social interaction. These behavioral changes are probably related to AChE decrease and indicate neurotoxicity. In the antioxidant enzymes, probably Al interacted with SOD, CTL, and GST resulting in an oxidative stress condition^32^. When the fish were exposed to Al and oils, the negative effects of the metal does not occur, suggesting a neuroprotector and antioxidant effect of the CBD- and THC-rich oils. In our previous work^8^, we observed that the CBD- and THC-rich oils were able to reduce oxidative stress and impair the acetylcholinesterase activity and behavioral biomarkers in the invertebrate animal model organism *Caenorhabditis elegans*; now we show similar effects in the zebrafish model.

To understand the kinetics of the compounds with Al, as a stressor, we quantified the concentration of THC and CBD in the aquaria water, using HPLC. Here we observed that the concentrations of THC in the THC-rich oil were higher than in the other oil and the same observation was for the CBD quantification in the CBD-rich oil. The presence of CBD in the THC-rich oil as well as the presence of THC, in the CBD-rich oil, is normal once for the oil extraction they use specific strains of *Cannabis sativa* which produces different amounts of each compound. To produce a THC-rich oil (APEPI) they use a transgenic strain of *Cannabis*, that produces a higher amount of THC compared to other cannabinoids. In this way, the higher production of THC does not completely avoid the production of CBD and other cannabinoids and it can explain the presence of other compounds in lower concentrations in a specific rich oil^33^.

An interesting result observed was the reduced concentration of THC and CBD in the groups treated with the oils + Al. In plants, terpenoids are produced to protect them against oxidative stress, toxicants, and physical predation^34^. Many of the terpenoids have a great antioxidant effect also in animals^35^. Both THC and CBD have considered specific terpenoids called cannabinoids and they can interact with toxic compounds reducing the toxicity^33^. In our study, we observed that in the presence of Al, both terpenoids (THC and CBD) had the concentration reduced in the aquarium water. It means that had occurred an interaction between the compounds and the Al which probably leads to the conjugation of the compounds for neutralizing the Al-toxicity. In the body, CBD and THC may work in a similar way reducing the toxicity induced by hazardous compounds^36^. In addition, the fish observed an increase in the antioxidant enzymes which suggests an increased oxidative resistance of the body to pro-oxidative compounds such as Al.

Our results show in the test of SPT that the Al reduced the interaction with the conspecifics. When Al is applied concomitantly with the THC-rich oil the social interaction is re-established. Related to behavior, a preserved behavioral repertoire is very important for species perpetuation^37^. Behavioral alterations are responses that came from internal (physiological) and external (environmental) interactions. The zebrafish (*Danio rerio*) is a social specie, that needs to interact with its conspecifics^38^. These findings in our study show the deleterious interaction of the Al in social behavior an important biomarker even for species perpetuation as well as for physiological and neural normal function. However, CBD- and THC oils would help in these observed changes.

The impact on cholinergic signaling and behavior was in part reversed by the THC-rich oil once the social preference was reestablished after THC administration. However, the anxiety-like pattern was not reversed after THC- or CBD-rich oils administration. Taken together, reduced social interaction and increased anxiety-like patterns exert a clear deleterious effect. Both observed behaviors originated from central nervous alterations such as cholinergic signaling. Another important behavioral biomarker is the anxiety pattern, which in humans is marked by intense, excessive, and persistent worry and fear of everyday situations^39^. In fish, this feeling-like condition is defined by reduced locomotion and reduced exploratory behavior^40^. A possible explanation for this discrepancy in the re-establishment of the social behavior but not the anxiety-like behavior after THC-rich oil administration might be due to the different behavioral paradigms used in these two experiments. THC is already known by causing anxiogenic effects when presented in high doses^41,42^, and the observed effect of increased anxiety-like behavior is related to anxiogenic episodes. However, the social behavior reestablishment might be related to the CBD presence even in low concentrations of this oil other studies have shown that at low concentrations of CBD^41,43^. In addition, CBD at low concentrations is responsible for decreasing the aggressiveness in mammalians by activating the 5-HT1A and CB1 receptors^10^, which can be linked with increased social behavior. In the environment, the resumption of the natural behavior of the species to gather in shoals is very important since the shoals have a clear advantage of protection, search for food, and reproductive success^44,45^. On the other hand, the anxious behavior observed here (staying at the bottom) seems to be the clearest defense strategy when one is alone without even seeing the conspecifics, such as the situation imposed for the NTT assessment.

Cholinergic signaling is responsible for learning, and locomotion, and is important in several processes, such as memory, neuronal development, and participation in the reward system^46^. Even more alterations in central systems are observed to understand and explain different disorders^47^. Al is considered a neurotoxic compound once is related to cholinergic damage in animals ^15^. In animals, Al interacts with AChE reducing its activity and causing neurotoxicity^48,49^. AChE is responsible for the breakdown of the synaptic ACh in the synaptic cleft. Reductions of this enzyme are responsible for longer allowance of the neurotransmitter ACh agonizing the cholinergic receptors^15^. This increased period of receptor agonization is responsible for a hyperexcitation of the cholinergic nervous system leading to individual changes and finally death. In our study, Al exposure reduced AChE accordingly to findings in the literature but just the THC-rich oil could reestablish the normal AChE activity.

In the central nervous system, several neurotransmitters seem to be interacting among different nervous systems. Both tested major compounds present in the oils are important neurotransmitters of the endocannabinoid system (ES). The THC and CBD are linked to agonizing the receptors CB1 and CB2 from the ES. The distribution and function of each one differ while the receptor CB1 is found predominantly in the central nervous system, as well as in the lungs, liver, and kidneys, the CB2 receptor is expressed mainly in the immune system and in hematopoietic cells^50^. Cannabinoids are synthesized on demand in the body or acquired exogenously. Neurotransmitters will be released upon activation by a process called retrograde neurotransmission. In this process, membrane receptors signal calcium channels, which cause cannabinoids to bind to receptors, generating a cascade of reactions, such as inhibition of adenylate cyclase, decrease in cyclic AMP levels, and opening of potassium channels, which will decrease the transmission of signals that will generate an inhibition of the release of neurotransmitters, such as GABA and glutamate^51^.

Overall, AChE reduced activity seems to be linked just with social preference alterations. It suggests that the observed change in social preference is reestablished by AChE modulation. As it was mentioned, the cholinergic nervous system is responsible for learning, cognition, and memory, for fish recognize themselves and the conspecific seems to be linked with cognition. In this way, the modulation of AChE activity is linked with the reestablishment of functional and favorable conditions for cognition. Regarding the anxiety-like observed profile, which was not reconstructed after oils administration it seems to be linked with another pathway of interaction. Generalized anxiety disorder is linked to a disbalance among the main neurotransmitters dopamine, serotonin, and glutamate^39^. The interaction between the endocannabinoid system and other neurotransmitters is still not well understood, but here we observe that even though cannabis oils can improve the cognition of fish it does not seem to improve a clear profile for reducing the anxiety induced by neurotoxicity.

At least, another important biomarker that is considered the basis of many diseases is the antioxidant system. The antioxidant system is composed of two groups with antioxidant proprieties called non-enzymatic antioxidant systems, composed of compounds with antioxidant activities e.g., carotenoids, flavonoids, phenols, anthocyanins, polyphenols, and others^52^. The second group is composed of enzymes with antioxidant activity such as superoxide dismutase (SOD), catalase (CTL), and glutathione-S-transferase (GST). These enzymes work to reduce toxic compounds to less toxic or non-toxic compounds seeking to reduce the deleterious effect of free radicals on the cell^53^.

We show that the exposure to Al alongside the THC- and CBD-rich oils increased the activity of SOD and that this increase was enough to avoid cellular damage once the CTL in these same groups was not increased. SOD and CTL work in a tandem manner, while SOD reduces the superoxide anion (O_2_^-^) to hydrogen peroxide (H_2_O_2_), the enzyme CTL uses the H_2_O_2_ produced by SOD to reduce until water and oxygen^54^. Other ways of H_2_O_2_ are presented in the animal body and can actuate right before or after CTL such as glutathione peroxidase (GPx)^55^. Here, the increased SOD and the non-increase in CTL represent that the produced amount of H_2_O_2_ produced was transformed by another pathway and finally indicates that probably the oils were able to avoid cellular damage. Differently from this, in the group treated with Al was no observed increase in SOD activity, but the enzyme CTL was largely increased. The activation of the CTL indicates that Al continuously produced H_2_O_2_ which can be dangerous for cells. The non-activation of SOD in the group treated just with Al might be linked with metal inhibition of the SOD. This enzyme has a metallic center that interacts with other metals such as Al, iron, copper, and zinc^32^.

The enzyme GST is responsible for the detoxication of toxic compounds from the cells and the body. It plays an important role in adding glutathione groups to lipophilic compounds transforming them into hydrophilic facilitating the elimination from the body^56^. In our study was observed that Al increased the GST activity highlighting the toxic pattern of Al on cells. When Al is applied with THC- and CBD-rich oils the GST activity is reduced characterizing a cellular protector effect of the oils.

## Conclusion

In our study, we observed that Al is responsible for neurotoxicity, especially causing AChE decrease. The main effect of Al is related to reduced social ability and anxiety-like patterns. The testes oil THC- and CBD-rich have an important role in AChE reestablishment and social ability reacquisition. In addition, both oils exert an outstanding effect on antioxidant enzyme modulations with the re-establishment of the SOD and CTL after Al exposition. The activity of GST was also well modulated indicating that the oils played a crucial role in cellular damage avoidance. However, the oils do not change the impaired anxiety-like behavior that looks to be linked to other central signaling pathways and needs to be well investigated in the next studies. Finally, the oils have a protective effect and might be used with proposals for neurological and antioxidant impairment avoidance.

## Material and methods

### Study strategy and experimental design

**Figure 8:**
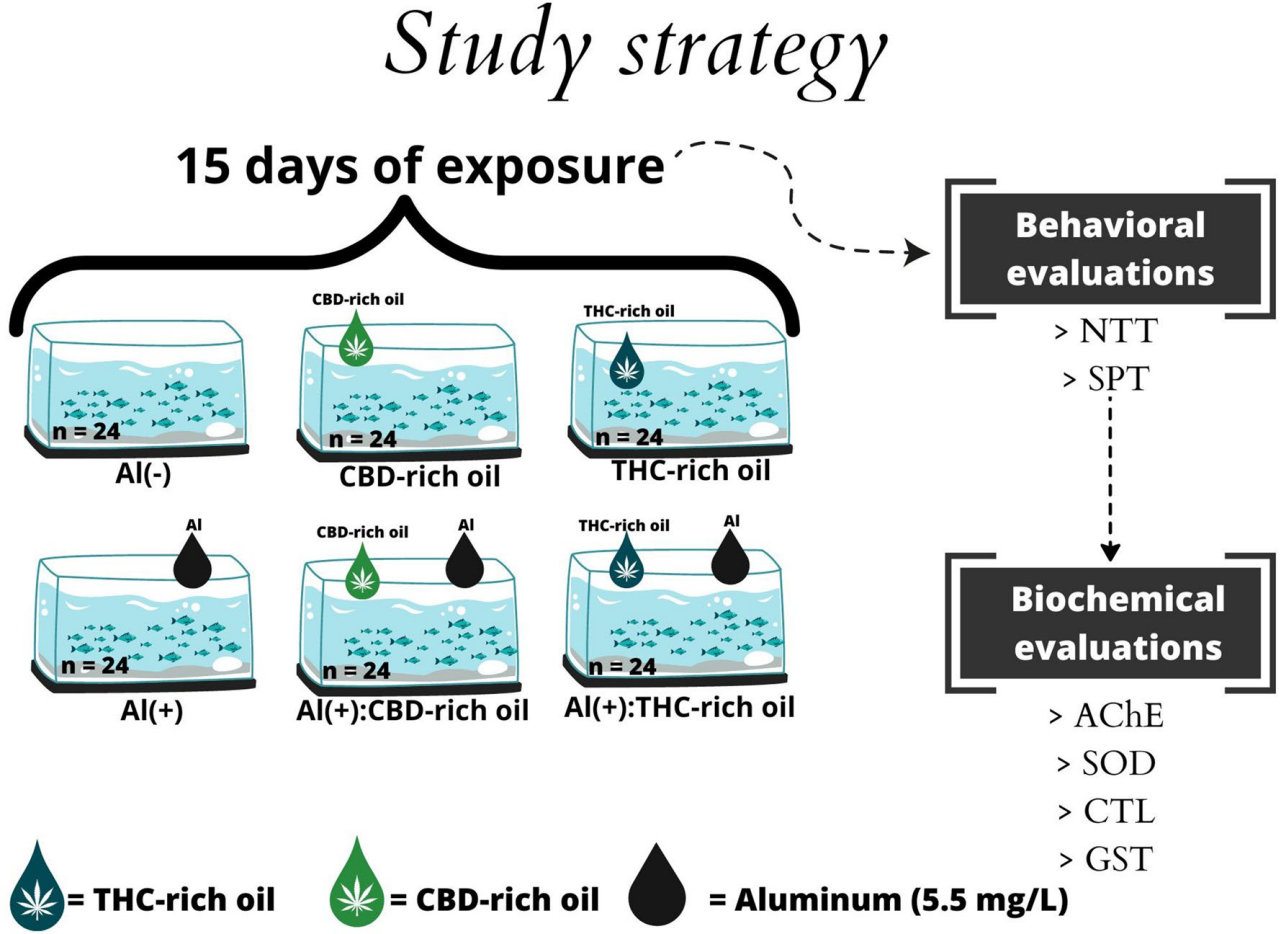
Study strategy. www.canva.edu.

Aiming to investigate whether CBD- and THC-based oils protect the brain from the toxic effects of Al, we used adult zebrafish as an animal model organism. Operationally, 144 fish were divided into 6 groups which were exposed to Al (5.5 mg/L) and with or without CBD- and THC-based oils (Fig. 8), and then behavioral biomarkers, antioxidant system biomarkers, and cholinergic signaling were evaluated.

The concentrations of CBD- and THC oils were established according to a previous work published by our group^8^. In addition, the Al concentration of 5.5 mg.L^-1^ was used according to Bortoli et al., (2018), which was known to induce neurotoxicity. In this way, the following six groups were composed: vehicle, Al(−); CBD-rich oil, Al(−); THC-rich oil, Al(−); vehicle and Al(+); CBD-rich oil and Al(+); and THC-rich oil and Al( +) (Fig. 8). Fish were exposed in the respective groups for 15 days (chronic exposure) and then were behavioral and biochemical evaluated.

### Ethical note

This study is under protocol number 1806240222 following the Conselho Nacional de Controle de Experimentação Animal (CONCEA) and was approved by the Ethics Commission for Animal Use (CEUA) at the IFRS, Campus Sertão/RS, Brazil. In addition, all methods are reported by ARRIVE guidelines (https://arriveguidelines.org).

### Fish husbandry and oils compositions

We used a population of 240 mixed-sex (1,1, male: female), adult, wild-type zebrafish (0.2–0.6 g) with a body length between 1.5 and 2.5 cm, from heterogeneous breeding stock and kept in an artificial photoperiod (12 h, 12 h dark: light), biological filtering, and constant aeration at a density of 2 fish·L^−1^. The dechlorinated tap water used in the control and exposed groups was maintained at 26 ± 2 °C, pH of 7.0 ± 0.2, with dissolved oxygen levels at 6 ± 0.5 mg·L^−1^, total ammonia levels below 0.6 mg·L^−1^, total hardness at 50 ± 5 mg·L^−1^, and alkalinity at 40 ± 3 mg·L^−1^, CaCO_3_. Fish were fed twice a day with commercial flaked food (Alcon Basic, Alcon, Camboriú, Brazil).

The oils were donated by APEPI—RJ (Support for Medicinal Cannabis Research and Patients), a non-profit initiative that aspires to fund study and the personal cultivation of Cannabis for therapeutic objectives. As the oils received by donated and the extraction protocols were not delivered by APEPI, they were employed for analysis on identical forms that they are sold for therapeutic goals. The vehicle used was ultrapure olive oil, also obtained by the APEPI–RJ and employed as a control treatment. These oils have the same composition as we also described^8^.

### Behavioral analyzes

All behavioral tests were video recorded using a Logitech C525 and analyzed with ANYmaze® tracking software (Stoelting Co., USA). For the three behavioral tests, we used a glass aquarium (20 × 20 × 25 cm [w × h × d]) filled with 7 L of water with the same characteristics as the experimental aquarium. To minimize interference, caution was observed to ensure that the animals were in the same tank region during all behavioral tests. All tests were performed between 06:30 a.m. and 06:30 p.m. for 3 consecutive days. Three behavioral tests were conducted in triplicate. The time of each parameter was strictly controlled to guarantee the standardization of the protocols. After the fish were euthanized, the homogenate was prepared for biochemical tests.

#### Novel tank test (NTT)

The NTT was conducted according to a previously described methodology^57^ (n = 24). We scored four key behavioral parameters: distance traveled (m), latency to enter the upper zone (s), time in the bottom zone (s), and time in the upper zone (s).

#### Social preference test (SPT)

The social preference was evaluated as described by Gerlai et al., ^44^ (n = 24). In this test, the time in the conspecific segment (s), the time in the empty segment (s), and the latency to enter the conspecific segment (s) were determined.

After behavioral tests, fish were desensitized to ice and were euthanized by cutting the spinal cord. Brain tissue was removed for biochemicals evaluations as following described.

### Biochemical analyzes

#### Acetylcholinesterase activity (AChE)

Twenty-four fish per group were separated into eight groups with three fish each (8 pools). The three brains (pool) of exposed and euthanized fish were directly extracted from the cranium and homogenized in an ice bath with 60 vol of potassium phosphate buffer (TFK 11 mM) using a Potter homogenizer. The hydrolysis speed of acetylthiocholine (8 mM) was determined in a reaction buffer (11 mM TFK, pH 7.0, and 0.22 mM DTNB)^58^. Protein-containing samples (15 µg) were incubated for 5 min at 35 °C. Afterward, absorption readings were taken at a wavelength of 412 nm for 5 min (at 30-s intervals). AChE activity was expressed as µmol ACh h^-1^·mg protein^-1^. All tests were performed in triplicates. Protein content was measured^59^ for all the samples and used for the calculation of the final activity of the enzymes.

#### Determination of superoxide dismutase (SOD), catalase (CTL), and glutathione-S-transferase (GST) activities

After exposure and euthanasia, the skull cap and brain were removed from the head, and so were the gills of the same fish. These three fish (one pool) were homogenized in 1.7 mL of Tris HCl (pH 7.0, 50 mM) for 1 min in a Potter mixer on an ice bath. Subsequently, it was centrifuged at 7,000 rpm for 10 min at 4 °C. The supernatant was collected for use in the enzymatic tests. The GST activity was performed as previously described ^60,61^. CAT activity was measured as described previously^62^. SOD activity was measured as previously described^63,64^.

### Delta-9-tetrahydrocannabinol (THC) and cannabidiol (CBD) determination by HPLC

THC and CBD analyte quantification were determined in the water as described by Tamagno et al., (2022)^47^ with some adaptations when the fish remained for the duration of exposure. The extraction was performed using QuEChERS Extraction (Agilent Technologies, catalog number 5982-0755) and QuEChERS Dispersive kits (Agilent Technologies, catalog number 5982-4950).

At the beginning of the experiment, right after the preparation of the water for exposition, we collected 15 mL of water from all groups in triplicate. The samples were immediately transferred to a Falcon tube (50 mL), 15 mL of acetonitrile (ACN) solvent was added for extraction, and the solution vortexed for 1 min. Thereafter, QuEChERS extraction salts were added, vortexed, and subsequently centrifuged for 5 min at 4000 × *g*. The supernatant (6 mL) was applied to the purification columns supplied in the Dispersive kit. The tube was vortexed for 1 min and then centrifuged for 5 min at 4000 × *g* before 2 mL of the supernatant was transferred to vials for chromatography.

Chromatographic analysis was performed using the Shimadzu high-performance liquid chromatography (HPLC) system composed of two quaternary pumps, an autosampler (model 20ACHT), and a diode array detector (DAD) (model SPD-M20A) and a C18 (ODS) reverse phase (150 mm × 4.5 mm) column. All modules were controlled using Software Lab Solutions.

Under these chromatographic conditions, 20 µL of each sample, composed of ACN/H_2_O (65/35) in the mobile phase, was injected into the isocratic module at a flow of 0.7 mL.min^-1^. The running time was 15 min, and the temperature was kept at 60 °C with a 20 µL sample injection and a retention time of 10 min. Quantification was performed by external standardization, and the standard curve was constructed with seven points with concentrations ranging from 25 mg·mL^-1^ to 150 mg·mL^-1^ of CBD or THC in ACN. Furthermore, an R^2^ of 0.944/158 was obtained for the equation (y = 811.13 × X + 9.46432e + 007). The identification was performed using readings obtained at 280 nm in the CLAE-DAD against the retention time of the analytes. The final concentration was expressed as mg·mL ACN^-1^.

### Statistics

The data of the three replicates were analyzed using two-way ANOVA (with the presence (+) or absence (−) of Al as independent factors); followed by Tukey's posthoc test, depending on the normality of the data (assessed by the Kolmogorov–Smirnov test). The graphs were constructed using Graph Pad Prism 8.0.1 Software (GraphPad Software 2365 Northside Dr. Suite 560 San Diego, CA 92108). Each graph is represented by the mean ± standard error.

### Ethical approval

This study was conducted under the guidelines of the National Council for the Control of Animal Experimentation (CONCEA) and was approved by the Ethics Commission for Animal Use (CEUA) at Instituto Federal do Rio Grande do Sul – campus Sertão, RS, Brazil (Protocol number 1806240222).

## Data Availability

The data that support the findings of this study are available from the corresponding author upon reasonable request.
